# Emerging evidence for the use of colchicine for secondary prevention of coronary heart disease

**DOI:** 10.5694/mja2.51488

**Published:** 2022-04-08

**Authors:** Stefan M Nidorf, Jamie Layland, Philip C Robinson, Sanjay Patel, Peter J Psaltis, Peter L Thompson

**Affiliations:** ^1^ Genesis Care Perth WA; ^2^ Heart and Vascular Research Institute Harry Perkins Institute of Medical Research Perth WA; ^3^ Monash University Melbourne VIC; ^4^ University of Queensland Brisbane QLD; ^5^ Royal Brisbane Hospital Brisbane QLD; ^6^ Royal Prince Alfred Hospital Sydney NSW; ^7^ University of Sydney Sydney NSW; ^8^ Royal Adelaide Hospital Adelaide SA; ^9^ Vascular Research Centre Heart Health Theme South Australian Health and Medical Research Institute Adelaide SA; ^10^ Sir Charles Gairdner Hospital Perth WA; ^11^ University of Western Australia Perth WA

**Keywords:** Coronary artery disease, Anti‐Inflammatory agents, Cardiovascular agents

Colchicine is an inexpensive new treatment for coronary heart disease that is both safe and effective in select patients

Cardiovascular disease imposes a major burden on Australians and the Australian health care system. Due to campaigns to reduce smoking and the widespread use of effective lipid‐lowering therapy, there has been a significant decline in the death rate from cardiovascular disease over several decades.^1^ However, nearly 600 000 patients are hospitalised each year with cardiovascular disease, at a cost to the community of over $4 billion in 2018–19.^1^ Patients with coronary heart disease face an ongoing risk of cardiovascular events even when their lipid‐lowering and antithrombotic therapy is optimal. Thus, to reduce morbidity in these patients, there is a need for doctors to employ additional therapies that are effective, safe, readily available and cost‐efficient for this purpose. In the past decade, increasing evidence has accrued suggesting that there are cardiovascular benefits associated with adding colchicine 0.5 mg daily to lipid‐lowering and antithrombotic therapy for secondary prevention of coronary heart disease.^2^

## Rationale for colchicine in coronary heart disease

The rationale for trialling colchicine in patients with coronary heart disease relates to the known importance of inflammation as a driver of the atherosclerotic process, and the known anti‐inflammatory effects and long term safety of colchicine.^3^ Emerging evidence that formation of cholesterol crystals within the arterial wall can incite an inflammatory process akin to gout, which transforms atheroma into atherosclerotic plaque, has further strengthened the rationale for its use.^4^ To date, the only other anti‐inflammatory agent with proven benefits in coronary heart disease is canakinumab, a specific inhibitor of interleukin‐1β (IL‐1β). In the CANTOS trial, the cardiovascular benefits of canakinumab were found to be modest.^5^ Because canakinumab treatment was associated with an increased risk of life‐threatening infection and came at a very high cost, the US Food and Drug Administration (FDA) did not approve an application for its use for secondary prevention of atherosclerosis.^6^ These results and the FDA decision raised questions as to whether anti‐inflammatory therapy could ever be employed safely and at reasonable cost in patients with coronary disease.^6^, ^7^

In contrast to canakinumab, colchicine is a widely available, inexpensive agent that has broad anti‐inflammatory effects. It is most avidly taken up by leukocytes, where its ability to bind tubulin affects the production of numerous cytokines, including IL‐1β.^8^ Long term colchicine at doses of 0.6–1.2 mg daily has been used for decades in a range of diseases, and is FDA‐approved for the prevention of acute inflammatory flares in patients with familial Mediterranean fever and gout.^9^

## Efficacy of colchicine in coronary heart disease

Evidence supporting the cardiovascular benefits of colchicine 0.5 mg once daily in patients with coronary heart disease now includes two large prospective randomised placebo‐controlled multicentre trials, LoDoCo2 (the second Low Dose Colchicine trial for secondary prevention of cardiovascular disease)^10^ and COLCOT (Colchicine Cardiovascular Outcomes Trial),^11^ which collectively included over 10 000 participants followed for up to 5 years. These trials demonstrated that colchicine improves disease‐free survival by reducing the risk of myocardial infarction, ischaemic stroke, and ischaemia‐driven revascularisations by 25–30%. Similar benefits out to 2 years were also demonstrated in the COPS (Colchicine in Patients with Acute Coronary Syndrome) trial.^12^ These benefits, which are similar to those achieved with statins, were seen in patients with both chronic and recently unstable coronary disease atop of usual therapy. Thus, these trials lay the foundation for colchicine to be adopted as an adjunct therapy for secondary prevention of coronary heart disease in the same way as the 4S (the Scandinavian Simvastatin Survival Study) and CARE (Cholesterol and Recurrent Events) trials established the efficacy of statins in fewer than 8000 patients.^13^

The benefits of colchicine appear consistent for secondary prevention in a range of patients at risk of further cardiovascular events, including those with and without a history of hypertension, diabetes and past coronary revascularisation. Moreover, the benefits of colchicine appear to be independent of other therapies, including statins, angiotensin‐converting enzyme inhibitors, and antithrombotic therapy.^10^, ^11^, ^12^, ^14^

## Safety of long term low dose colchicine

Low dose colchicine is well suited in patients with coronary heart disease, as it does not cause or aggravate bleeding or affect blood pressure, it is not pro‐arrhythmic and it does not cause or affect renal or liver function.^15^ Further, the cardiovascular trials confirm that long term low dose colchicine is safe. Compared with placebo, colchicine 0.5 mg once daily did not increase the incidence of new or fatal cancer, serious or fatal infection, myotoxicity or neutropenia, and was not associated with a risk of serious interactions with drugs commonly used in patients with coronary disease, including high dose statins.^10^

Some concern was raised that, in the LoDoCo2 trial, the fewer cardiovascular deaths in patients assigned to colchicine were counterbalanced by an increase in the number of all other forms of non‐cardiovascular death.^10^ However, this post hoc observation in a small number of patients was not statistically significant and was not seen in the COLCOT trial or demonstrated in subsequent meta‐analyses in patients with and without cardiovascular disease.^14^, ^16^

Reassuringly, pharmacodynamic studies confirm that colchicine 0.5 mg daily does not raise serum levels above the upper limit of safety when used in patients without advanced renal or liver disease, or when used concomitantly with many common medications.^15^ Nonetheless, as with any long term treatments, the cardiovascular benefits of low dose colchicine need to be weighed against any non‐cardiovascular risks. Although no serious concerns have been identified, ongoing trials should be undertaken to provide further surety about any unforeseen risks.

## Tolerance and safe prescribing of low dose colchicine

As with statins, colchicine is associated with intolerance in up to 10% of patients. Unlike statin intolerance, which generally occurs late, gastrointestinal intolerance to colchicine typically declares early. Early intolerance is not a marker of systemic toxicity. Diarrhoea is usually mild, often transient, invariably ceases when therapy is stopped, and can be potentially avoided by introducing colchicine at 0.25 mg daily before graduating to 0.5 mg daily. In patients without early intolerance, long term tolerance is favourable. Although myotoxicity was not encountered in the large clinical trials, persistent muscle pain should prompt a check of renal function and creatine kinase levels; and if creatine kinase is elevated, both colchicine and statins should be ceased.^9^, ^15^, ^16^, ^17^

Colchicine should be avoided in patients with advanced renal disease (creatinine clearance < 30mL/min) or advanced liver disease and in those with haematological conditions associated with neutropenia, and it should not be prescribed concomitantly with clarithromycin, antifungal or antirejection therapy.^9^, ^15^, ^16^, ^17^

## Guideline and regulatory approval

The use of colchicine 0.5 mg daily in addition to lipid‐lowering and antiplatelet therapy is not yet approved for coronary heart disease in Australia; therefore, clinicians need to be aware that they would need to prescribe it off‐label. However, it is now included in the guidelines for secondary prevention in select patients with atherosclerosis in Latin America^18^ and Europe,^19^ and has been approved for this purpose by Health Canada,^20^ where its use has been demonstrated to be cost‐effective (Box).

Box 1 **Updated guidelines related to the use of low dose colchicine for secondary prevention of cardiovascular disease (CVD) in other countries^21^**

**Table:** 

Jurisdiction	Recommendation	Details	Date	Level of evidence	Strength of recommendation
Latin America^18^	Latin American consensus on management of residual cardiometabolic risk	After myocardial infarction and patients with evidence of stable coronary disease, after treatment with statins, aspirin and ACEI/ARB	June 2021	IIb	B
Europe^19^	Guidelines on cardiovascular disease prevention in clinical practice	May be considered in secondary prevention of cardiovascular disease, particularly if other risk factors are insufficiently controlled or if recurrent cardiovascular disease events occur under optimal therapy	September 2021	IIb	A
Canada^20^	Health Canada	May be considered for the reduction of atherothrombotic events in adults with existing coronary heart disease in addition to standard therapies, including LDL cholesterol‐lowering and antithrombotic therapies	August 2021		

ACEI = angiotensin‐converting enzyme inhibitor; ARB = angiotensin II receptor antagonist; LDL = low‐density lipoprotein.

## Prescribing colchicine for secondary prevention of coronary heart disease

The question now facing doctors in Australia is whether they should begin prescribing colchicine for their patients with coronary heart disease or wait a few more years for the results of new trials or wait even longer in the hope that novel anti‐inflammatory therapies being developed for atherosclerosis might be more effective than colchicine.

Given the lengthy process required for the development of novel therapeutic agents, it is likely that colchicine will remain the most effective, safest, most widely available, and least expensive anti‐inflammatory therapy for secondary prevention of coronary disease for some time to come. Thus, considering the updated guidelines for secondary prevention of coronary disease in other countries, it is our opinion that Australian doctors should become familiar with low dose colchicine and confident in prescribing it to suitable patients with cardiovascular disease even as ongoing trials continue, because its cardiovascular benefits come at little risk and at low cost.

## Competing interests

Aspen Pharmacare Australia provided colchicine and matching placebo for the Australian arm of the LoDoCo2 trial.

## Provenance

Not commissioned; externally peer reviewed.
